# Acute stress reaction, depression anxiety stress, and job withdrawal behavior in non-frontline pediatric nurses during the pandemic: a cross-sectional study

**DOI:** 10.3389/fpsyt.2023.1123445

**Published:** 2023-05-12

**Authors:** Xu Yi, Cao Jing, Ma Meimei, Xie Jianhui, Hu Jihong, Xiang Ding, Zhu Lihui

**Affiliations:** ^1^Department of Rehabilitation, Hunan Children’s Hospital, Hunan, China; ^2^Department of Nursing, Hunan Children’s Hospital, Hunan, China; ^3^International Affairs Department, Hunan Children’s Hospital, Hunan, China

**Keywords:** pediatric nurses, COVID-19, acute stress reaction, depression, anxiety, stress, job withdrawal behavior

## Abstract

**Backgrounds:**

The COVID-19 pandemic has brought an unprecedented healthy crisis to people worldwide. It is crucial to assess the psychological status of non-frontline nurses. More attention to the mental and physical health of non-frontline nurses during a public health emergency is necessary for a full understanding of the implications. Therefore, this study aims to investigate the factors that influence the acute stress reaction of non-frontline pediatric nurses during the COVID-19 pandemic.

**Methods:**

This study aimed to explore factors associated with acute stress reactions of non-frontline pediatric nurses in Hunan province during the COVID-19 pandemic. This was a cross-sectional design. Five hundred eighteen pediatric nurses from Hunan province, China, completed the Stanford Acute Stress Reaction Questionnaire (SASRQ), Depression Anxiety Stress Scales-21 (DASS-21), and Job Withdrawal Behavior Scales (JWB). Multiple linear regression analyses and Pearson’s correlation were used to analyze the results.

**Results:**

The mean scores of DASS-21, JWB, SASRQ were 1.443 ± 0.500, 1.601 ± 0.544, and 1.858 ± 0.805, respectively. Stress, anxiety, depression (three sub-dimensions of DASS-21), JWB, monthly income and department were the major predictive factors for SASRQ (Adjusted *R*^2^ = 0.579, *p <* 0.001). Pearson’s correlation showed that the mean score of SASRQ was positively correlated with JWB, DASS-21, and all its dimensions (*p* < 0.01).

**Conclusion:**

The study indicated that the SASRQ was greater with higher levels of DASS-21 and JWB. It revealed an acute stress reaction in non-frontline pediatric nurses and recommends more focus on the factors influencing the SASRQ.

## Introduction

Acute stress reaction refers to individuals who have a physical and psychological reaction that occurs 2–28 days after experiencing or witnessing a traumatic event (1).

When this reaction exceeds a certain intensity and/or duration, it will have a negative impact on the individual’s social functioning, interpersonal interactions, and emotions, and will develop into an acute stress disorder (2). It is undeniable that there has been a great effort to develop guidelines for the treatment of acute stress disorder and post-traumatic stress disorder in countries around the world (3). However, according to relevant research data (4) around 65% of men and 50% of women reported having experienced at least one traumatic event. Notably, relatively high rates of non-remission were reported among some special populations (e.g., military populations, firefighters) (3). Increasing attention is being paid to the impacts of public health disaster on humans as both society and technology develop (5). Like SARS and COVID-19, they have brought about an unprecedented health crisis.

Nurses, as the largest group of healthcare providers, play a crucial role in reducing the spread of the virus and caring for severely-ill patients during the COVID-19 pandemic (6). At the same time, nurses exposed to stressful factors related to the high-risk working environment, such as contact with patients and heavy workload, may also exhibit physical and psychological stress. Pediatric nurses, in particular, have a significantly more stressful and difficult job than nurses in other departments due to the young age of children, poor expressive skills and the rapid changes of disease. A study by Jin and Hu (7) found that a series of acute stress responses occurred in nurses during the COVID-19 pandemic, including cognitive changes, altered mood, physical changes, or behavioral changes. Many previous studies have focused on the related effects of acute stress events on the mental and physical health of frontline nurses during the COVID-19 pandemic (8, 9), studies have rarely addressed non-frontline pediatric nurses. During the pandemic, the frontline staff is defined as those participants who support fever clinics, isolation units, or Wuhan city. Compared with frontline, non-frontline nurses are not in contact with COVID-19 patients or their body fluid, nor were they isolated in a hotel. Yet, they need to undertake a huge nursing workload which put them at potential risk of infection. For example, before the highly suspected patients were diagnosed, non-frontline nurses could only take secondary protection for patients according to hospital infection standards. Undoubtedly, this phenomenon has increased non-frontline nurses’ fear and psychological burden of getting risk of infection. Previous reports have shown high rates of adverse mental health symptoms among the general population and vulnerable groups during past outbreaks of infectious diseases. In a literature meta-analysis by Krishnamoorthy Y et al. (10), the pooled prevalence of mental disorders such as post-traumatic stress disorder (27%), depression (26%), anxiety (26%), and stress (34%) in the Chinese population during the covid-19 pandemic was shown. According to the psychological stress theory of Lazarus and Folkman (11), individuals will generate a series of cognitive, emotional, and physiological responses to respond to sudden crises after experiencing major stressful events. If coping styles fail, corresponding physiological dysfunction and psychological distress will occur. Although job withdrawal behavior among nurses has been underreported in the studies available to date, the results of a previous study (12) by a research team showed that under a major public health event, a sudden increase in stressors at work tends to elicit various stress responses in nurses, which in turn further motivate individuals to become dissatisfied with their work environment and ultimately lead to withdrawal behaviors. Moreover, there are few studies on the relationship between anxiety, depression, stress, job withdrawal behavior and stress reactions, yet it is necessary to explore the nature of the association between these factors and acute reactions for researchers and medical policy makers to identify future research needs and drive policy decisions. Therefore, we used the cross-sectional survey to assess the relationship between acute stress reaction, depression anxiety stress and job withdrawal behavior among the non-frontline pediatric nurses in China.

## Methods

### Study population

A total of 518 pediatric nurses from three districts in Hunan province in central China completed online questionnaires. Participants were not able to skip sections or individual items. To protect their privacy, all data was anonymized. The inclusion criteria were: to volunteer for the study and sign the informed consent form, and registered nurses engaged in clinical work for ≥1 year. The exclusion criteria included nurses from the COVID-19 unit and nurses on leave or study leave for more than 1 month during the survey period.

### Instruments

Four questionnaires were used to collect the data:

1. Demographic information, including gender, age, marital status, hospital level, frequency of night shifts each month, monthly income, education (highest), and department.
2. The Stanford Acute Stress Reaction Questionnaire (SASRQ) was used to evaluate the acute stress response. It was compiled by Cardeña (13), and the Chinese version was translated by Jia (14). It has been widely used in the nursing population. The scale involves 30 items (5 dimensions) including the dissociative symptoms (10 items), avoidance symptoms (6 items), re-experiencing symptoms (6 items), hyper-arousal symptoms (6 items), and impairment in social functioning (2 items). Each item was assessed with a six-point Likert scoring system (1–Never to 6–Always). The range of scores is from 0 to 150 with higher scores indicating a more severe symptom (cut off score 40). More than 40 as moderate, and more than 57 as severe. Cronbach’s 𝜶 of the scale was 0.974. This study investigated the acute stress level of individuals during the pandemic. Therefore, the ‘acute stress event’ of the original scale was changed to ‘COVID-19’.
3. The Depression Anxiety Stress Scales-21 (DASS-21) was used to screen the general population or nurses’ psychological status. It was compiled by Lovibond (15) and the Chinese version was translated and revised by Taouk et al. (16). The scale is comprised of three separate subscales, involving 21 items, with seven items each for depression, anxiety, and stress subscales. Each item was assessed by a four-point Likert scoring system (1–Never to 4–Always). The total score is calculated from the sum of the scores of the 21 items and then multiplied by two. Higher scores indicate stronger negative emotions. Cronbach’s 𝜶 of each dimension was 0.910, 0.913, and 0.921, respectively.
4. The Job Withdrawal Behavior (JWB) scale was used in this study Lehman and Simpson (17). It includes 12 items. Each item is rated on a five-point Likert scoring system (1–Never to 5–Always). Higher scores indicated more severe withdrawal behavior. Cronbach’s 𝜶 of the scale was 0.902.

### Data collection

Data were collected using the online questionnaires platform Questionnaire Star Platform (Changsha Ranxing Information Technology Co., Ltd). The questionnaire was distributed through the social platform WeChat. The collection was between March 2 to April 1, 2020. This period was the early stage of the COVID-19 epidemic. The questionnaire was available at https://www.wjx.cn/wjx/design/previewmobile.aspx?activity=66301334, and issued by the primary researcher, with instructions regarding the aim and significance on the first page. Informed consent was requested on the first question: ‘Do you agree to participate in this investigation voluntarily?’. Participants were required to click ‘Yes’ to the next question. Participants scanned the two-dimensional code using their smartphones and filled out the questionnaires online; the time for answering was 15 min. To ensure the quality of the questionnaire and the data quality, a WeChat username was restricted to answer only once and each questionnaire was reviewed by two trained investigators. A convenience sample was available and used for this study. Thus, no sample size calculations were made (18).

### Data analysis

Prior to hypothesis testing, all data were examined to determine missingness, identify extreme values, and confirm that the data structure met analytic assumptions. All analyses were conducted using IBM SPSS Statistics version 23.0. Continuous data were categorized into categorical variables. For the descriptive statistics, the number, percentage, mean and standard deviation were calculated. Independent samples t-test was used for independent group comparisons. Differences among group means were tested by *t*-test or ANOVA *F*-test. Pearson’s correlation test was used to define the relationship between variables (SASRQ, DASS-21, and JWB), and multiple linear regression analysis was performed for multivariate analysis. All tests were two tailed, and *p* < 0.05 was considered significant.

## Results

### Test of common method bias

Harman’s one-factor test was used to assess the common method bias of the data. The results showed that the maximum factor variance interpretation rates was 34.56%, which is below the cut-off value of 40% (19). Hence, common method bias was not a serious problem in this study.

### Subject data and univariate analysis of SASRQ

A total of 518 subjects completed the questionnaires. These included 15 males and 503 females, with an age range 19–58 years. The majority (59.3%) of subjects were in the age range 26–35 years, 18.0% were aged 19–25 years, 20.1% aged 36–45 years, and 2.7% aged 45–58 years. Marital status was categorized into single (25.7%), married (71.4%), and divorced (2.9%). Hospital level included primary hospital (9.1%), secondary hospital (2.1%), tertiary hospital (85.9%), and others (2.9%). Monthly income was divided into 3 groups (<4,000 RMB, 4,000–8,000 RMB, and >8,000 RMB). In total, 13.9% of subjects had monthly incomes of less than 4,000 RMB, 59.7% had monthly incomes of 4,000–8,000 RMB, and 26.4% had monthly incomes of more than 8,000 RMB. The overall incidence of SASRQ was 68.7%. Of these, 11% at age below 25, 41.9% at age 26–35, 14.5% at age 36–45,and 1.4% at age 45 or over. Details are summarized in Table 1.

**Table 1:** Subject data and univariate analysis of SASRQ (*n* = 518).

Demographic variables	Group	*n* (%)	Mean ± SD	*t*/*F*	*P*
Gender	Male	15 (2.9)	1.878 ± 0.947	0.098	0.922
	Female	503 (97.1)	1.857 ± 0.801		
Age (year)	~25	93 (18.0)	1.805 ± 0.859	0.733	0.533
	~35	307 (59.3)	1.879 ± 0.803		
	~45	104 (20.1)	1.879 ± 0.786		
	>45	14 (2.7)	1.593 ± 0.594		
Marital status	Single	133 (25.7)	1.842 ± 0.831	0.197	0.821
	Married	370 (71.4)	1.858 ± 0.793		
	Divorced/Separated	15 (2.9)	1.980 ± 0.914		
Hospital level	Primary hospitals	47 (9.1)	1.583 ± 0.544	4.662	0.003**
	Secondary hospital	11 (2.1)	1.349 ± 0.520		
	Tertiary hospital	445 (85.9)	1.909 ± 0.829		
	Others	15 (2.9)	1.564 ± 0.585		
Frequency of night shifts/Month	1 ~ 2 night shifts/month	42 (8.1)	1.712 ± 0.826	2.532	0.040*
	3 night shifts/per month	20 (3.9)	1.827 ± 0.716		
	4 night shifts/month	179 (34.6)	1.818 ± 0.787		
	≥5 night shifts/month	117 (22.6)	2.058 ± 0.902		
	Non-night shift	160 (30.9)	1.798 ± 0.735		
Monthly income	<4,000 RMB	72 (13.9)	1.643 ± 0.721	3.342	0.036*
	4,000–8,000 RMB	309 (59.7)	1.871 ± 0.805		
	>8,000 RMB	137 (26.4)	1.940 ± 0.832		
Education (Highest)	Technical secondary school and below	5 (1.0)	1.533 ± 0.549	1.077	0.358
	College degree	90 (17.4)	1.745 ± 0.706		
	Bachelor degree	403 (77.8)	1.889 ± 0.830		
	Master’s degree and above	20 (3.9)	1.817 ± 0.745		
Departments	Emergency & intensive system	143 (27.6)	2.050 ± 0.924	4.578	0.004**
	Surgical system	102 (19.7)	1.714 ± 0.783		
	Internal system	161 (31.1)	1.768 ± 0.698		
	Out-patient system	112 (21.6)	1.871 ± 0.766		

^*^p < 0.05, ^**^p < 0.01.

Significant differences were found among nurses in different levels of hospitals, night shifts, monthly income, and departments on the level of acute stress response (Table 1). Specifically, SASRQ scores (1.909 ± 0.829) were the highest among nurses working in the tertiary hospital (*p* < 0.01). The scores (2.058 ± 0.902) for nurses who worked ≥5 night shifts were significantly higher than other nurses (*p* < 0.05). Moreover, the SASRQ scores (1.940 ± 0.832) of nurses with high income were significantly higher than other nurses (*p* < 0.05). For different departments, the scores (2.050 ± 0.924)of nurses who worked in emergency and intensive care departments had significantly higher scores compared with nurses working in other departments (*p* < 0.01).

### Correlation between SASRQ, DASS-21, and JWB and its scores

Table 2 shows that all the correlations among variables were at significant levels (rs = 0.60–0.93, *p* < 0.01). The scores of SASRQ, DASS-21, and JWB were 1.858 ± 0.805, 1.443 ± 0.500, 1.490 ± 0.544, 1.429 ± 0.512, 1.410 ± 0.489, and 1.601 ± 0.544, respectively.

**Table 2:** Correlation between SASRQ, DASS-21 (*n* = 518).

	SASRQ	Stress	Anxiety	Depression	JWB
SASRQ	1				
Stress	0.736**	1			
Anxiety	0.731**	0.927**	1		
Depression	0.722**	0.922**	0.899**	1	
JWB	0.539**	0.604**	0.611**	0.620**	1

**p < 0.01.

### Multivariate analyses of SASRQ scores

A multiple regression analysis was conducted to examine whether demographic variables including hospital level, frequency of night shifts/month, monthly income, and department predicted SASRQ scores. The dummy variables setting was used for unordered classification variables in independent variables (see Table 3).

**Table 3:** Variables assignment.

Independent variables	Assignments
Hospital level	Primary hospitals = 1; Secondary hospitals = 2; Tertiary hospital = 3; Others = 4
Frequency of night shifts/Month	1 ~ 2 night shifts/month = 1; 3 night shifts/month = 2; 4 night shifts/month = 3; ≥5 night shifts/month = 4; Non-night shift = 5
Monthly income	<4,000 RMB = 1; 4,000–8,000 RMB = 2; >8,000 RMB = 3
Departments	Emergency & intensive system = 1; Surgical system = 2; Internal system = 3; Out-patient system = 4

Table 4 shows the results of the multiple linear regression analysis. The results suggest that monthly income of 4,000–8,000 RMB yuan and departments of emergency and intensive system are the major predictive factors for SASRQ.

**Table 4:** Multiple linear regression analysis of influencing factors of SASRQ (*n* = 518).

Independent variables	*B*	*SE*	*β*	*t*	*P*
Constant	0.052	0.184	-	0.281	0.779
Hospital level (with ‘others’ as reference)	-	-	-	-	-
Primary hospitals	0.007	0.160	0.026	0.449	0.653
Secondary hospitals	0.006	0.214	0.001	0.026	0.979
Tertiary hospital	0.087	0.156	0.038	0.557	0.578
Frequency of night shifts/Month (with ‘Non-night shift’ as reference)	-	-	-	-	-
1 ~ 2 night shifts/month	−0.0119	0.092	−0.040	−1.288	0.198
3 night shifts/per month	−0.090	0.062	−0.053	−1.448	0.148
4 night shifts/month	−0.080	0.125	−0.019	−0.639	0.523
≥5 night shifts/month	0.023	0.071	0.012	0.318	0.751
Monthly income (with ‘>8,000 RMB yuan’ as reference)	-	-	-	-	-
<4,000 RMB yuan	−0.151	0.092	−0.065	−1.638	0.102
4,000 ~ 8,000 RMB yuan	−0.123	0.055	−0.075	−2.236	0.026
Departments (with ‘Out-patient system’ as reference)	-	-	-	-	-
Emergency and intensive system	0.198	0.075	0.110	2.620	0.009
Surgical system	0.025	0.077	0.013	0.327	0.744
Internal system	0.010	0.071	0.006	0.135	0.893

In these comparisons, other, non-night shift, >8,000 RMB yuan, and the out-patient system as reference categories for hospital level, frequency of night shifts/month, monthly income, and departments, respectively. Β = standardized regression coefficient; B = unstandardized regression coefficient. Multiple linear regression model: *R*^2^ = 0.592, adjusted *R*^2^ = 0.579, *F* = 45.473, *p* < 0.001.

## Discussion

Related research from SARS in 2003 showed that medical staff not only experienced more stress during the epidemic but also posttraumatic stress after the outbreak (20). Therefore, mental health issues among nurses arising from fighting COVID-19 are also a huge challenge. Interestingly, a study in China showed that the traumatization scores of non-frontline nurses were higher than frontline nurses (21). The possible reason is that most frontline nurses volunteered and were provided with additional professional training. Another cross-sectional study from China compared the frontline nurses and non-frontline showed that the non-frontline nurses were more stressed than the front-line nurses (22). The reasons might lie in several aspects. For example, a unit of non-frontline nurses is used to prepare for receiving new infectious diseases. However, they are not provided with separate accommodations to ensure that lived separately from their families. The majority of nurses are concerned that their families and friends would be infected. Therefore, we should pay attention to the psychiatric status of non-frontline nurses. The study measured the socio-demographic characteristics of SASRQ, as well as the relationship between JWB and DASS-21 with SASRQ among non-frontline pediatric nurses in China. To our knowledge, few studies have investigated the relationship between DASS-21, JWB, and SASRQ among non-frontline pediatric nurses during the COVID-19 pandemic. Our main findings showed that a positive correlation was found between SASRQ, JWB, DASS-21, and all its sub-dimensions (*p* < 0.01). In addition, monthly income and departments were the major predictive factors for SASRQ (Adjusted *R*^2^ = 0.579, *p* < 0.001).

### Acute stress reaction of non-frontline pediatric nurses during the COVID-19 pandemic

Our findings show that the majority of nurses are experiencing SASRQ. In the study of Shahrour et al. (23) from Jordan, incidence of SASRQ among nurses was 64%. This is roughly consistent with the specific observation collected herein.

However, there is a systematic review across 19 studies showed that the prevalence of acute stress was 31% (24). The reason may be that the majority of our sample are women (97.1%). Some scholars pointed out that a higher prevalence of SASRQ was found among women (25). Also, the mean SASRQ score was 1.858 ± 0.805, which indicates a moderate to severe level of non-frontline nurses in our study. Similar results were found in the past in a meta-analysis study (26). The prevalence of mental health symptoms of participants in Wuhan, the epicenter of the COVID-19 crisis in China, was significantly lower than that in Non-Wuhan samples (*p* = 0.038). This may be due, in part, to the fact that governments and institutions pay more attention to the physical and psychological needs of frontline nurses. Meanwhile, psychological support could help these population-frontline nurses, for instance, in a study by Zhang et al. (27), psychological support was provided by psychotherapists; normalized training prior to rescue work (6), or some psychosocial support to improve the mood through telephone conversations with family and friends. This notion is also substantiated by a study showing that non-frontline nurses faced with stressful situations without enough psychological support developed various acute stress reactions over time (28). Consequently, hospital managers should pay more attention to this situation in the future. With the various public health emergencies frequently occurring, in order to minimize possible complications, it is necessary to learn from this experience.

### Influencing factors of acute stress reaction among non-frontline nurses in Hunan province

The results showed that SASRQ scores had a positive correlation with DASS-21 scores and its three dimensions (stress, anxiety, and depression). This is consistent with findings in a study by Zheng et al. (29). Results of studies by Trautmann et al. (30), Wheaton et al. (31) showed that negative emotions (e.g., stress, anxiety, and depression) were related to the susceptibility of emotional contagion in individuals. This suggests that individuals who experience higher emotion contagion will have a greater stress response to traumatic events, such as the global pandemic. Furthermore, individuals who are prone to negative emotions, such as attention loss and anxiety, have a greater risk of nursing errors and it may affect nurses’ and patients’ health and the quality of clinical care. These data are similar to the results from certain developing countries, such as Brazil (32). Therefore, it would be beneficial for nurses to control negative emotions, and finding ways to release stress and anxiety will help reduce the level of SASRQ scores. The present study also found a significant moderate correlation between JWB and SASRQ (*r* = 0.539, *p* < 0.001). Previous research has indicated that SASRQ scores have a significant positive relationship with JWB (12). Withdrawal is one of the important concepts in the field of developmental psychology and psychiatry, and it is often manifested as job withdrawal in the workplace. JWB refers to the behavior of avoiding work tasks when individuals face pressure and dissatisfaction with their working conditions and roles, or the hidden negative behavior of flouting work rules (12). Compared with the negative emotional experience of job burnout, job withdrawal places greater emphasis on the negative behavior of individuals at work. Therefore, the substantial consequences of job withdrawal are often more destructive and harmful. Psychological research suggests that positive coping strategies are beneficial to health (33). Therefore, it is essential to encourage individuals to use positive coping mechanisms to deal with acute stress events.

In this study, the univariable analysis revealed significant differences between SASRQ scores (*p <* 0.05) among monthly income and department. The multiple linear regression demonstrated that participants with higher SASRQ scores were significantly more likely to be in the *>* 8,000 RMB group than in the 4,000 *~* 8,000 RMB groups (*p <* 0.05), which is inconsistent with the results of previous studies. Previous studies (34) found that nurses could alleviate burnout or emotional exhaustion by increasing external available resources (e.g., monthly income). Furthermore, the results of a study from a developing country showed a non-existent relationship between income and emotional exhaustion (23). The reason is that some scholars have pointed out that what affects psychology is the perception of income insufficient, rather than actual income (35). A more reasonable explanation is that nurses in our study were from different hospital. The income of recruited nurses in each hospital depending on the hospital-level. Nurses of some hospitals with a higher grade run a great risk of infection, and thus, a higher SASRQ score for higher paid nurses than for those with lower salaries. According to previous study results, income is an important predictor of the JWB for nurses. Xiong et al. (12) found nurses who reported low levels of income tended to experience more JWB, which also increases the possibility of acute stress reaction. Nevertheless, more research is needed to further explore the nature of the relationship between income, JWB and SASRQ. A study by Li et al. (36) found that department is an important factor affecting the level of acute stress reaction. Nurses in the emergency and intensive care departments are more likely to elicit an acute stress reaction, in comparison with nurses in other departments. This may be due to the pressure faced by nurses in intensive care compared with other departments, as they are dealing with severely ill patients, which makes the nurses anxious and under increased pressure. Besides, this may be due to the high turnover of patients and the increased risk of infection from COVID-19. Therefore, it is critical to study the factors that relate to mental health and emotions with COVID-19 and to understand how nurses respond to the pandemic.

This study showed that the hospital level and the frequency of night shifts do not predict SASRQ scores during the pandemic. As speculated, nurses at different hospital levels have to respond to patients with suspected COVID-19, varying exposure risks and work intensity, as well as different levels of nurses’ knowledge, which all affect SASRQ scores. Regarding the frequency of night shifts, frequent night shifts lead to sleep deprivation, which further stimulates the negative emotions of nurses within the working environment according to the previous study (37). However, our findings indicated the opposite. The reasons for this are worth exploring. According to previous researches in many countries, it had been observed that COVID-19 has negatively affected the mental health of individuals. Such as, China (26), Africa (38), Spain (8), Latin America (9), et al. quarantine adherence increased the presence of mental health disorders (32). Similarly, Hawryluck et al. reported that some SARS-exposed quarantines experienced emotional problems (39). Especially for those pediatric nurses who care for children in the hospital, it is also an important factor affecting mental health. This is an interesting possibility worthy of further study.

Limitations of the present study include the small sample size and that the study was conducted in one province of China. Second, a self-designed demographic questionnaire was used for linear regression, which may bring limitations. Besides, since it was a cross-sectional survey, the causal relationship cannot be confirmed. Furthermore, the generalizability of our findings to all nurses is limited since these findings are pertinent to pediatric nurses.

## Conclusion

The findings of this study revealed that DASS-21, JWB, departments, and monthly income were predictive factors of SASRQ scores. In addition, the results showed a correlation between SASRQ, JWB, DASS-21, and all its sub-dimensions. Future studies should explore additional underlying factors that cause acute stress reactions, and focus on the psychological status of pediatric nurses to provide better care for patients.

## Data availability statement

The raw data supporting the conclusions of this article will be made available by the author, without undue reservation.

## Ethics statement

The studies involving human participants were reviewed and approved by The ethics committee of Hunan children’s hospital approved the study protocol and the approval number is HCHLL-2021-61. The patients/participants provided their written informed consent to participate in this study.

## Author contributions

XY and CJ draft of the manuscript and analysis the data. MM and HJ collect the data and analysis the data. XD and XJ correct the English grammar. ZL design the study and collect the data. All authors contributed to the article and approved the submitted version.

## Funding

This work was supported by International Talent Training Program of Hunan Children’s Hospital, Scientific Research of Hunan Children’s Hospital.

## Conflict of interest

The authors declare that the research was conducted in the absence of any commercial or financial relationships that could be construed as a potential conflict of interest.

## Publisher’s note

All claims expressed in this article are solely those of the authors and do not necessarily represent those of their affiliated organizations, or those of the publisher, the editors and the reviewers. Any product that may be evaluated in this article, or claim that may be made by its manufacturer, is not guaranteed or endorsed by the publisher.
